# DAZAP1 facilitates the alternative splicing of KITLG to promote multiple myeloma cell proliferation via ERK signaling pathway

**DOI:** 10.18632/aging.204326

**Published:** 2022-10-13

**Authors:** Yanyan Zhou, Shaohua Huangfu, Muxi Li, Chao Tang, Jinjun Qian, Mengjie Guo, Zuojian Zhou, Ye Yang, Chunyan Gu

**Affiliations:** 1 Nanjing Hospital of Chinese Medicine Affiliated to Nanjing University of Chinese Medicine, Nanjing, China; 2 School of Medicine and Holistic Integrative Medicine, Nanjing University of Chinese Medicine, Nanjing, China; 3 School of Pharmacy, Nanjing University of Chinese Medicine, Nanjing, China; 4 College of Artifical Intelligence and Information Technology, Nanjing University of Chinese Medicine, Nanjing, China

**Keywords:** multiple myeloma, DAZAP1, alternative splicing, KITLG, ERK

## Abstract

Multiple myeloma (MM) is an incurable plasma cell malignancy, in which alternative pre-mRNA splicing (AS) acts as one of the key transcriptome modifier. The Deleted in Azoospermia-Associated Protein 1 (DAZAP1) is a splicing factor that has been identified as an oncogene in multiple cancers, yet its role in MM proliferation remains unclear. We first analyzed MM clinical databases and found that MM patients with elevated DAZAP1 had a poor survival. Furthermore, we overexpressed DAZAP1 by lentiviral transfection and utilized siRNA silencing the expression of DAZAP1 in MM cells. DAZAP1 promoted MM cell proliferation *in vitro* and accelerated MM xenograft tumor growth *in vivo*. KEGG pathway enrichment analysis showed that ERK signaling pathway was activated in DAZAP1-OE MM cells. The analyses of RIP-seq and RIP-qPCR revealed that DAZAP1 activated alternative splicing of KIT proto-oncogene ligand (*KITLG*) mRNA. Further study validated that DAZAP1 increased ERK phosphorylation via modulating alternative splicing of KITLG mRNA to promote MM cell proliferation. In conclusion, we establish DAZAP1 as a tumor-promoting gene with therapeutic potential and provide mechanistic insights into targeting DAZAP1 as a new strategy for the diagnosis and treatment of MM.

## INTRODUCTION

Multiple myeloma (MM) is the second most common hematologic malignancy characterized by uncontrolled proliferation and accumulation of abnormal plasma cells in the bone marrow (BM), leading to end-organ destruction [1, 2]. It is generally manifested by renal failure, anemia, bone lesions, and hypercalcemia. MM evolves from monoclonal gammopathy of unknown significance (MGUS), an asymptomatic premalignant stage to overt plasma cell leukemia and extramedullary myeloma [3]. The current strategies for MM include maintenance therapy, novel chemotherapy drugs such as proteasome inhibitors and immunomodulators, autologous stem cell transplantation (ASCT), etc., [4]. These effective treatments have dramatically enhanced complete response, progression-free survival, and overall survival in MM [5]. Nonetheless, evolving into relapse and drug resistance remain major causes of morbidity and mortality for MM patients [6]. MM presents a complex pathogenesis because of clonal heterogeneity [7], thus it is particularly important to explore molecular prognostic markers for risk stratification. It is necessary to elucidate the mechanism of malignant proliferation and find new strategies for the treatment of MM.

Alternative splicing (AS) of eukaryotic transcripts is an essential component of gene expression whereby introns are removed and exons are assembled in diverse combinations [8, 9]. The mechanisms broaden the diversity of functional proteins from a limited number of genes, which ultimately generate proteins with distinct or even opposing functions [10]. RNA mis-splicing is not only capable of causing a vast repertoire of diseases, but also the fundament of occurrence and development of tumors. Increasing evidences indicate that AS changes are vital signatures for tumor progression and therapy [11]. AS is a sophisticated process, strictly managed by the spliceosome, which is composed of five small nuclear RNAs (snRNA U1, U2, U4, U5 and U6) and a large number of splicing factors (SFs) [12]. Among these SFs, two RNA SF families have been well-studied. One is the serine-arginine-rich SFs (SRSFs) and the other one is heterogeneous nuclear ribonucleoproteins (HNRNPs). SRSFs tend to bind to splicing enhancers, while HNRNPs primarily bind to splicing silencers [13]. Anczukow O et al. [14] found that elevated SRSF1 promoted proliferation and mammary epithelial cell transformation in breast cancer. Golan-Gerstl R et al. [15] investigated that overexpression of hnRNP A2/B1 could enhance the production of anti-apoptotic isoforms by AS in glioblastomas. Consequently, aberrant splicing constitutes an important source of novel cancer biomarkers. SFs affecting the splice site selection of spliceosomes may represent an attractive target for novel therapeutic agents.

DAZAP1 was initially identified as an important binding partner of DAZ (deleted in azoospermia) and DAZL (deleted in azoospermia like), required for not only spermatogenesis but also the normal growth and development of mammals [16, 17]. DAZAP1 is an RNA binding protein (RBP) expressed in various tissues, and participates in post transcriptional modifications, such as AS, nucleocytoplasmic transport and translation [18]. Many evidences have demonstrated that DAZAP1 plays a vital role in the regulation of numerous cancers. However, it is still unclear the role of DAZAP1 in MM proliferation. In present study, we explored the correlation between DAZAP1 expression and the outcomes of MM patients. Further work demonstrated that DAZAP1 modulated the ERK signaling pathway by regulating the AS of *KITLG* mRNA to promote MM proliferation. Our data suggested that DAZAP1 might be a promising marker and potential therapeutic target for MM.

## RESULTS

### High-expressed DAZAP1 is related to poor survival of MM patients

**Figure 1:**
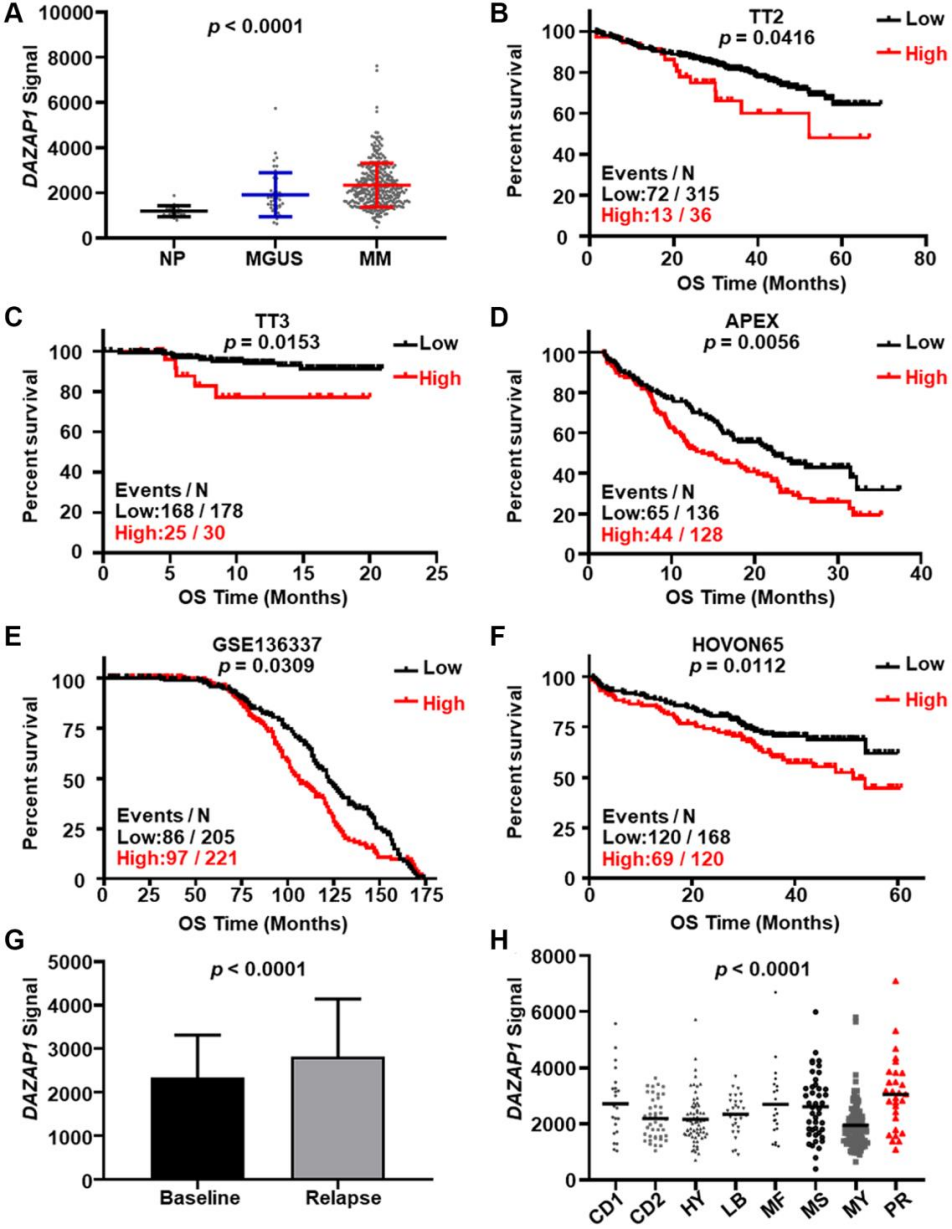
**Elevated DAZAP1 is associated with poor outcome of MM patients.** (**A**) DAZAP1 expression in different stages of MM from the GSE5900 dataset as shown in the graph. The signal level of DAZAP1 (226620_at signaling) was shown on the y-axis. The groups of normal plasma (NP, *n* = 22), monoclonal gammopathy of undetermined significance (MGUS, *n* = 44), and multiple myeloma (MM, *n* = 351) were sorted on the x-axis, respectively. (**B**–**F**) Kaplan-Meier analysis of overall survival (OS) divided by high and low DAZAP1 expression in TT2, TT3, APEX, HOVON65 and GSE136337 cohorts. The number of patients in the cohorts was 351, 208, 264, 426 and 288, respectively. (**G**) DAZAP1 expression was increased in relapsed patient samples relative to the first diagnosis samples. (**H**) A box-plot showed DAZAP1 expression in eight MM subgroups.

To evaluate the relevance of DAZAP1 to MM, we first analyzed the expression of DAZAP1 based on the GEP cohorts. Data from GSE5900 showed a significant increase of DAZAP1 in MM samples compared to the normal plasma cells (Figure 1A). After validating the four independent GEP cohorts included GSE2658, GSE9782, GSE19784 and GSE136337, we found that high expression of DAZAP1 were distinctly associated with poor survival of MM patients (Figure 1B–1F). In addition, we compared the DAZAP1 expression between baseline samples and relapsed samples and the result disclosed the relapsed MM patients had an enhanced expression of DAZAP1 (Figure 1G). Similarly, DAZAP1 expression in PR subgroup, the worst subgroup in MM with the high proliferation characteristic, was dramatically higher than another seven subgroups (Figure 1H). These findings above suggested that increased DAZAP1 expression was correlated with poor outcome of MM patients.

### Elevated DAZAP1 expression promotes MM cell proliferation *in vitro*

**Figure 2:**
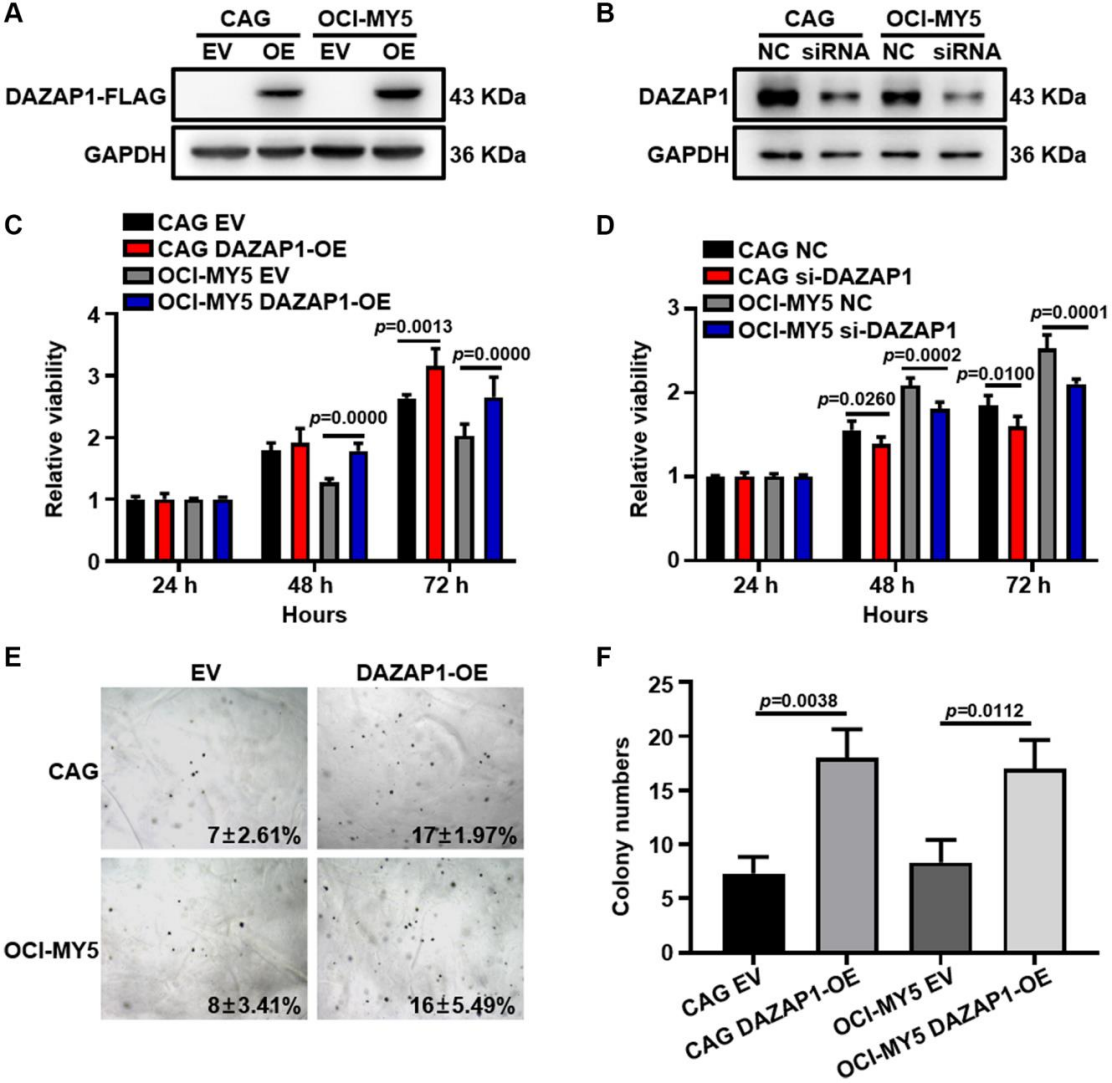
**Overexpression of DAZAP1 enhances the proliferative capacity of MM cells *in vitro*.** (**A** and **B**) DAZAP1 expression in DAZAP1-OE and siDAZAP1 MM cells were examined by WB analysis. (**C** and **D**) The proliferation rate of DAZAP1-OE and siDAZAP1 MM cells was assessed by CCK8 assay. (**E**) Soft agar colony formation assay revealed overexpressed DAZAP1 accelerating colony formation. (**F**) The histogram showed quantification of colony formation in soft agar. All data management and analysis were done using the GraphPad Prism 8.0 version. The *P* value was calculated with Student’s *t*-test, (^*^p < 0.05, ^**^p < 0.01, ^***^p < 0.001).

To further explore the role of DAZAP1 in MM cell proliferation, DAZAP1 was overexpressed stably in CAG and OCI-MY5 cells via lentiviral transfection while was knocked down using small interfering RNA (siRNA). The transfection efficiency was assessed using WB (Western blot) (Figure 2A and 2B, Supplementary Figure 1A and 1B). CCK8 assays showed that MM cell proliferation capacity was significantly enhanced in DAZAP1-OE cells compared to EV cells, while it was significantly suppressed in siDAZAP1 cells compared to NC (Negative control) cells (Figure 2C and 2D). In addition, the soft agar colony formation assay uncovered that CAG and OCI-MY5 DAZAP1-OE cells both had stronger long-term proliferative capacity (Figure 2E and 2F). Taken together, the results demonstrated that high-expressed DAZAP1 contributed to MM proliferation.

### DAZAP1 accelerates cell growth in MM murine xenograft model *in vivo*

**Figure 3:**
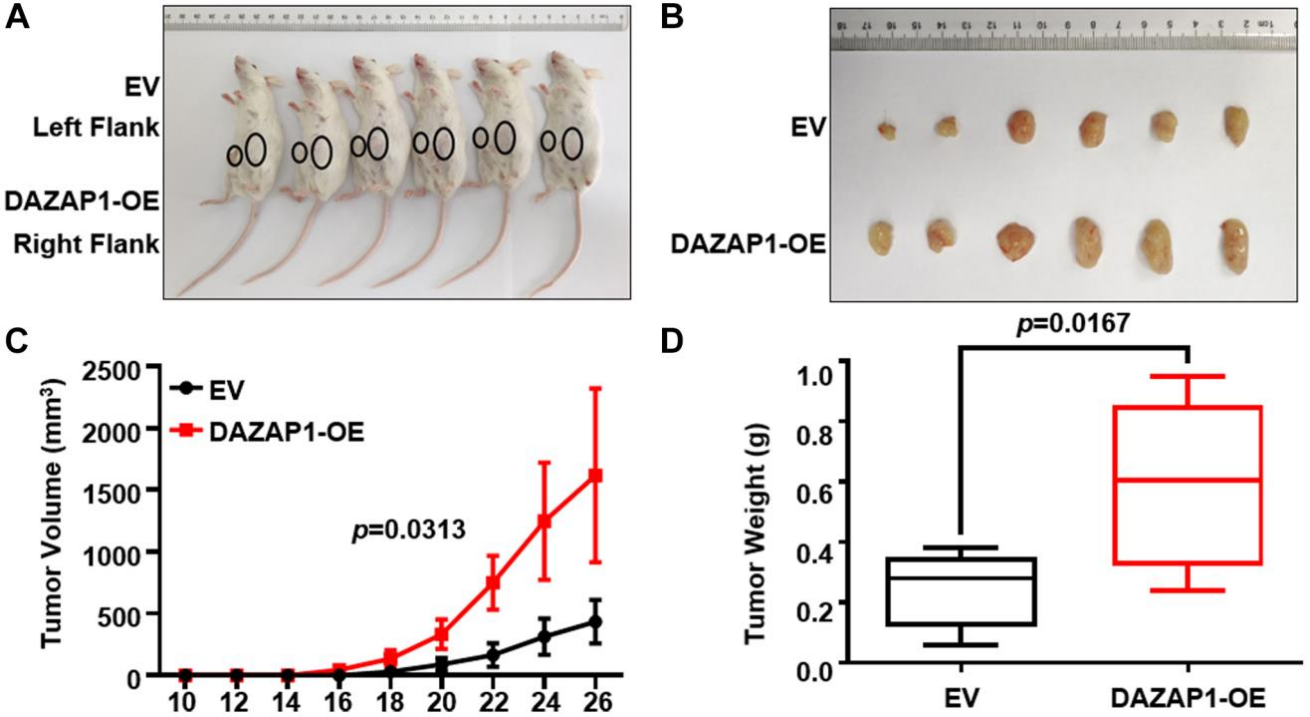
**Increased DAZAP1 is conducive to tumor growth in MM xenograft model *in vivo*.** (**A**) Photographic images of tumor burden mice were captured on day 26. (**B**) Subcutaneous tumors were gathered on the 26th day post grafting. (**C**) Growth curve of transplanted tumors. (**D**) Quantification of tumors weight from dissected tumors. All data are displayed as mean ± SD (^*^p < 0.05, ^**^p < 0.01, ^***^p < 0.001).

To further extend the proliferative function of DAZAP1 *in vivo*, CAG EV cells (left flank) and CAG DAZAP1-OE cells (right flank) were subcutaneously injected into NOD/SCID mice respectively. As depicted in Figure 3A and 3B, the tumors formed by CAG DAZAP1-OE cells were visibly larger than CAG EV counterparts. Congruously, both the volume and weight of CAG DAZAP1-OE tumors were also statistical significantly higher than those of CAG EV tumors (Figure 3C and 3D).

### DAZAP1 activates ERK signaling pathway in MM

**Figure 4:**
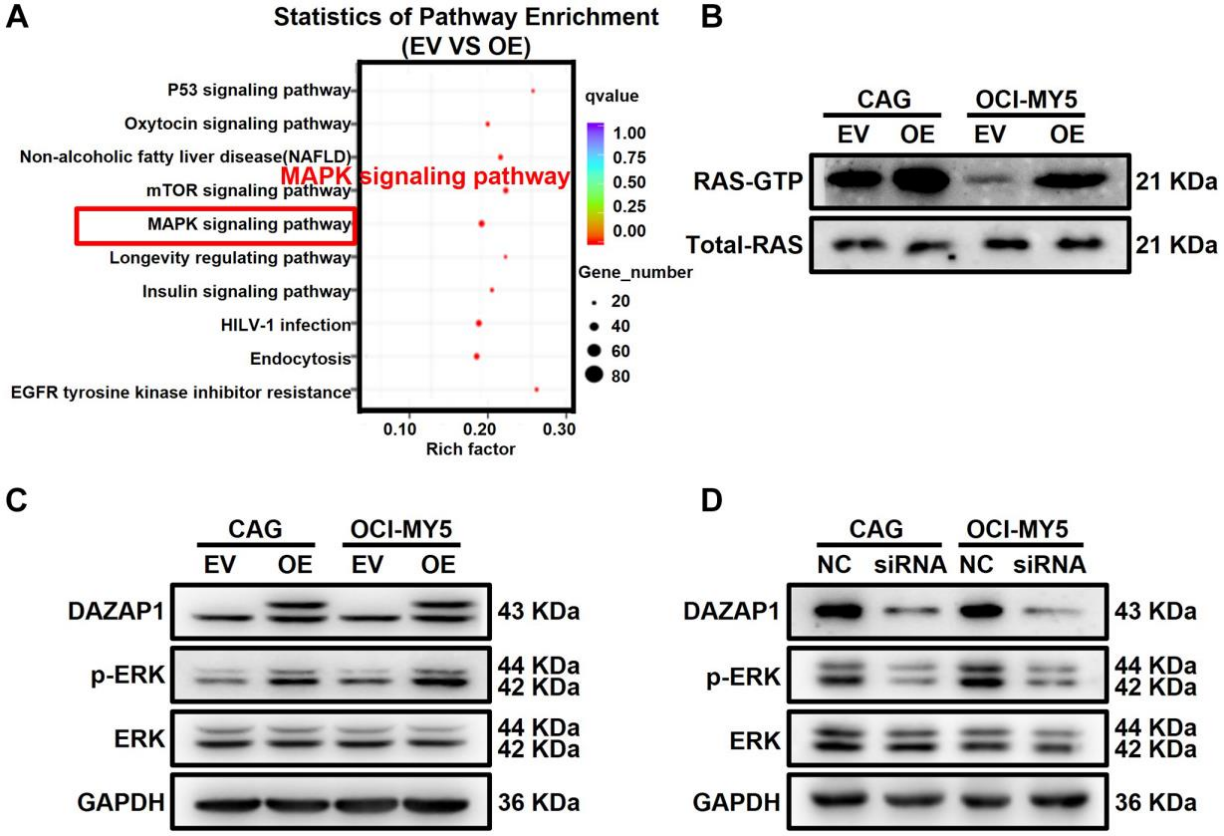
**DAZAP1 promotes the phosphorylation of ERK in MM cells.** (**A**) A bubble diagram of the top 10 KEGG pathways. In the bubble diagram, the vertical axis indicates the KEGG pathways and the horizontal axis represents the enrichment ratio. The sizes of the dots indicate the number of genes in the Gene Ontology term. (**B**) Active RAS Pull-down assay and Western blot showed the levels of active form RAS protein compared to total RAS protein. (**C** and **D**) WB test examined the phosphorylation level of ERK expression in DAZAP1-OE and siDAZAP1 MM cells.

To better understand the mechanism of DAZAP1 on modulating MM cell proliferation, RIP-seq assay was conducted in CAG EV and CAG DAZAP1-OE MM cells (GSE189240). KEGG pathway enrichment analysis was displayed as bubble diagram (Figure 4A). More genes were enriched in the MAPK signaling pathway which was identified as the potential signaling pathway involved in the regulation of DAZAP1 on MM cell proliferation. It has been reported that ERK signaling pathway plays an important role in MM cell proliferation. For instance, Hua Z et al. found that YTHDF2, an m6A reader, promoted MM cell proliferation via MAP2K2/ERK axis [19]. As a result, we intended to verify whether DAZAP1 promoted MM proliferation by activating the ERK signaling pathway. RAS can regulate the phosphorylation of ERK. Thus, we first detected the effect of DAZAP1 expression on RAS-GTPase activity by using an Active RAS Pull-Down and Detection Kit. The results showed that overexpression of DAZAP1 could remarkably increase RAS-GTPase activity (Figure 4B, Supplementary Figure 1C). As expected, WB assay indicated that overexpression of DAZAP1 increased ERK phosphorylation in MM cells, whereas knockdown of DAZAP1 effectively reversed the result. (Figure 4C and 4D, Supplementary Figure 1D and 1E).

### DAZAP1 activates alternative splicing of *KITLG* mRNA in MM cells

**Figure 5:**
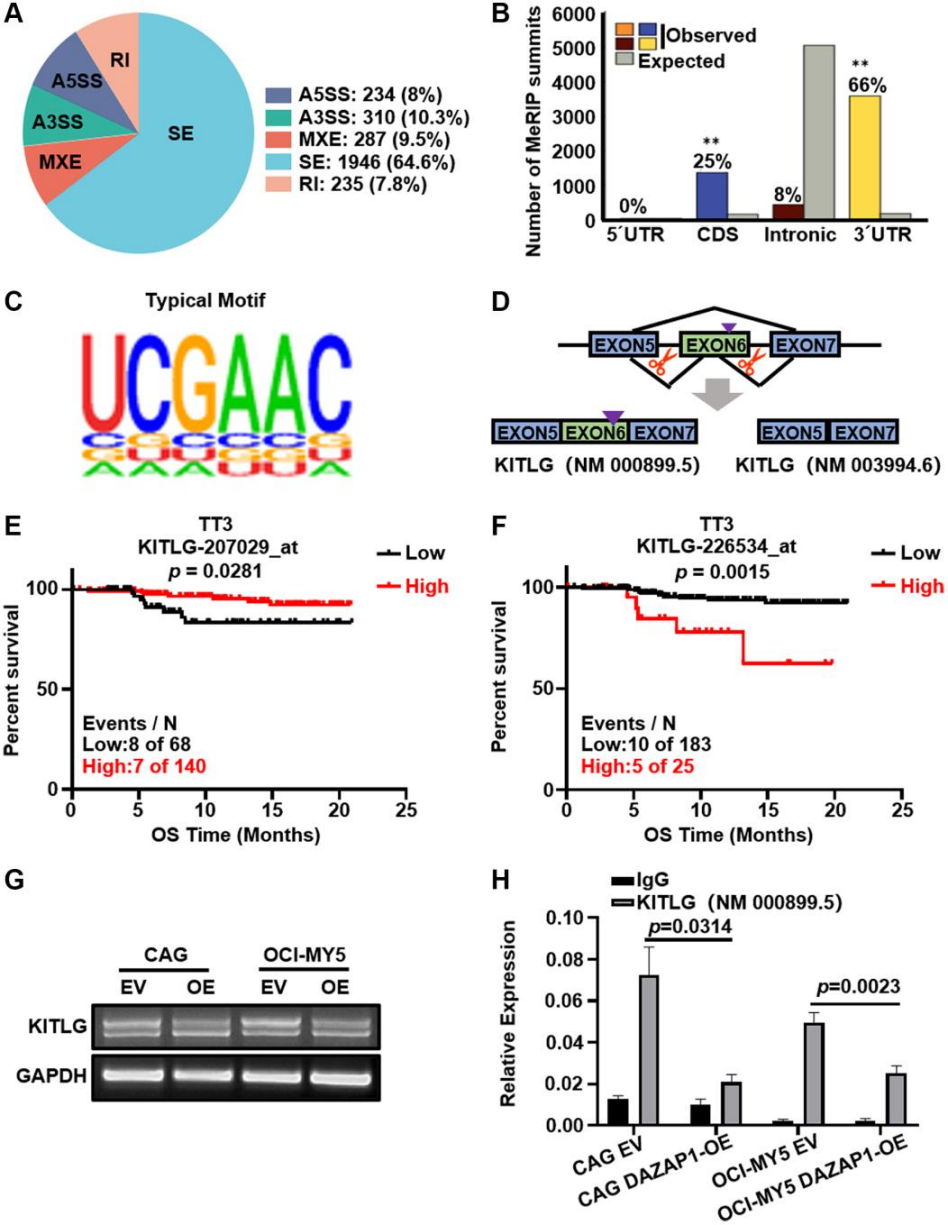
**DAZAP1 triggers alternative splicing of KITLG mRNA in MM cells.** (**A**) AS events were classified into five categories: skipped exon (SE), alternative 5′ splice site (A5SS), alternative 3′ splice site (A3SS), mutually exclusive exon (MXE), and retained intron (RI). (**B**) Binding of DAZAP1 was close to the 3′ splice site. (**C**) The typical motif on the peak-bound mRNA regions. (**D**) Schematic diagrams showed the alternative splicing of KITLG. (**E** and **F**) Two different probes of KITLG corresponded to different patient survivals in TT3 cohort. (**G** and **H**) RNA levels of different isoform of KITLG were tested by PCR and qPCR assays.

In order to further investigate the regulatory mechanism of DAZAP1 in MM, AS analysis by rMARTs defined thousands of DAZAP1-mediated AS events. As shown in the chart, skipped exon (SE) was the predominant type of AS event accounting for 64.6% of the total AS events, followed by alternative 3′ splice site (A3SS) and mutually exclusive exon (MXE). The remaining types of AS events, alternative 5′ splice site (A5SS) and retained intron (RI), showed lower frequencies. It is indicated that DAZAP1 principally modulated SE (Figure 5A). We further determined positional effects of DAZAP1 binding on cassette exons, and binding close to the 3′ splice site generally promoted exon skipping (Figure 5B). The typical motif of AS was shown in Figure 5C. We combined the analyses of RIP-seq with MM GEP cohorts to screen DAZAP1-regulated AS targets associated with MM progression. According to this strategy, *KITLG*, which skipped exon 6, was selected as the downstream target gene to further explore their contribution to MM progression. KITLG has two splicing isoforms, KITLG isoform2 (NM_000899.5) containing the primary proteolytic-cleavage site and KITLG isoform1 (NM_003994.6) lacking the primary proteolytic-cleavage site (Figure 5D). Furthermore, the probe 207029_at of KITLG was associated with favorable overall survival (Figure 5E). On the contrary, the probe 226534_at of KITLG was in connection with poor survival of MM patients in TT3 (Figure 5F). To evaluate the accuracy of variable splicing analysis, we carried out PCR to verify these two splicing isoforms of KITLG in MM. Additionally, overexpression of DAZAP1 resulted in a reduction of long transcripts. (Figure 5G). RIP-qPCR proved that DAZAP1 directly bound to endogenous KITLG isoform2 and promoted its splicing (Figure 5H).

### Aberrant splicing of KITLG contributes to the phosphorylation of ERK

**Figure 6:**
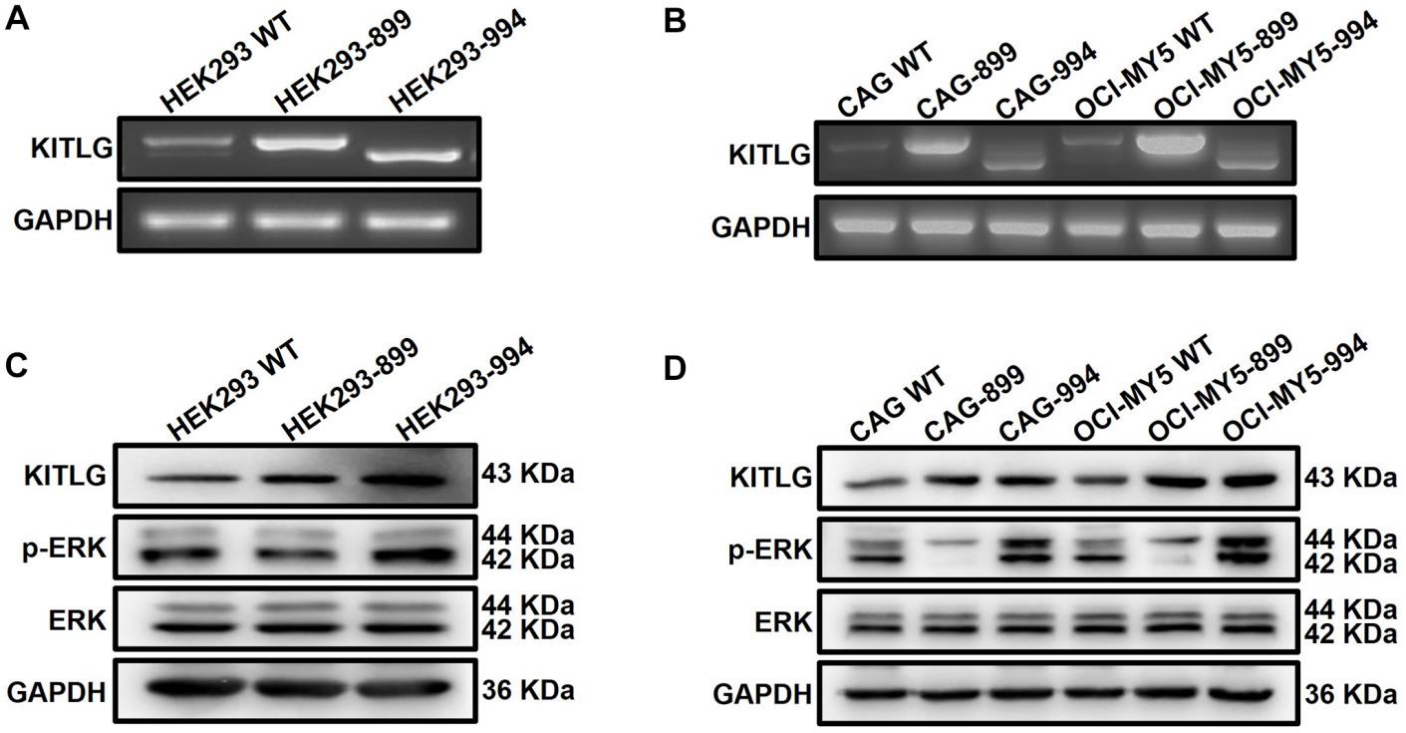
**Abnormal alternative splicing of KITLG activates the ERK signaling pathway.** (**A** and **B**) Agarose gel electrophoresis demonstrated the transfer efficiency on different isoforms of KITLG in both HEK293 and MM cells. (**C** and **D**) WB test verified that phosphorylated ERK expression was enhanced in KITLG isoform1-OE cells and downregulated in KITLG isoform2-OE cells.

The present study raised the possibility that DAZAP1 regulated the ERK signaling pathway through affecting the AS events of KITLG. To further validate this hypothesis, we conducted electroporation to transfect KITLG isoform1 and KITLG isoform2 into HEK293 cells separately. The transfection efficiency was visualized on 1% agarose gel electrophoresis and WB, and the phosphorylation levels of ERK were detected by WB. The results manifested that overexpression of KITLG isoform1 promoted the phosphorylation of ERK, while overexpression of KITLG isoform2 inhibited the phosphorylation of ERK (Figure 6A and 6C, Supplementary Figure 1F). The same conclusion was verified in MM cells (Figure 6B and 6D, Supplementary Figure 1G).

## DISCUSSION

MM remains an incurable hematologic malignancy, though significant improvements have been made in the molecular mechanisms of myeloma genesis, prognostication and treatment options in MM [20]. Our group is focused on exploring the innovative potential biomarkers to offer prognostic strategies for MM treatment. In present study, we investigated a novel target DAZAP1 in MM cell proliferation, which could promote AS of *KITLG* mRNA and facilitate the activation of ERK signaling pathway.

DAZAP1 is considered to be a multifunctional RBP localized in the nucleus, which has important regulatory roles in a variety of tumors, such as Wang Q et al. found that DAZAP1 promoted hepatocellular carcinoma proliferation by regulating ferroptosis [21]; Choudhury R et al. demonstrated that DAZAP1 integrated splicing control into MEK/ERK-regulated non-small cell lung carcinoma proliferation and migration [22]; Chen Y et al. reported that DAZAP1 integrated splicing control into TSC2-regulated autophagy in esophageal squamous cell carcinoma [23]. However, the role of DAZAP1 in MM has not been revealed. In present study, we found a close relationship between high DAZAP1 expression and poor outcome in MM patients. Further *in vivo* and *in vitro* proliferation experiments showed that increased DAZAP1 significantly promoted the proliferation of MM cells and accelerated the growth of MM xenograft tumors, whereas knockdown of DAZAP1 distinctly blunted the MM cell proliferation. Our findings fill a gap in the regulatory role of DAZAP1 on promoting the progression of MM. As an evolutionarily conserved RBP, DAZAP1 participates in several cellular processes, such as transcription, mRNA translation and RNA splicing. DAZAP1 modulates RNA expression by getting command of AS, mRNA stability and localization and translation efficiency. To date, accumulating data have shown that DAZAP1 is particularly associated with AS. Chen H et al. identified the regulatory regions where DAZAP1 bound and promoted the inclusion of Crem exon 4, Crisp2 exon 9 and Pot1a exon 4 [24]. The NF1 exon 37 and BRCA1 exon 18 mutations created new binding sites for DAZAP1 and caused skipping of the respective exons [25, 26]. Overexpression of DAZAP1 decreased the efficiency of *cox6c* pre-mRNA splicing, leading to the reduction of COX6C protein and the accelerated cell growth [18].

We employed RIP-seq method to further clarify the potential mechanism of DAZAP1 in MM. KEGG enrichment analysis indicated that the downstream targets of DAZAP1 were significantly involved in the MAPK signaling pathway. We screened 3,012 genes with differential AS mediated by DAZAP1, among which SE accounted for the majority beyond A3SS, MXE, A5SS, RI. Further experiment determined positional effects of DAZAP1 binding to cassette exons, and binding close to the 3′ splice site generally promoted exon skipping. Then we identified KITLG as the key downstream target of DAZAP1, which was involved in the activation of MAPK signaling pathway. KITLG is a ligand of the c-KIT, also known as a stem cell factor (SCF), which is involved in cell proliferation, differentiation, and stemness [27, 28]. Moreover, the probe 207029_at of KITLG was associated with favorable overall survival, on the contrary, the probe 226534_at of KITLG was related to poor survival of MM patients in TT3.

Owing to differential splicing, KITLG exists in two isoforms distinguished by a specific exon 6 encoding a proteolytic cleavage site. The variant carrying exon 6 encodes a soluble protein and the other one lacking exon 6 is a membrane-bound protein. Both isoforms have different impacts on the survival and proliferation of hematopoietic cell lines [29]. Recent findings of KITLG in chronic lymphocytic leukemia (CLL) indicate that KITLG is overexpressed in CLL B cells compared to healthy B cells, and the membrane-bound isoform plays a leading role [30]. KITLG helps c-Kit^+^ cancer stem cell (CSC) survival in selective culture conditions and promotes their canonical stemness properties, interestingly only the expression of membrane-bound SCF can be detected in ovarian tumor cells [31]. In this study, we assessed the expressions of two transcripts of KITLG and found the soluble isoform predominated in MM cells. Overexpressed DAZAP1 caused the exclusion of KITLG exon 6 to produce a short KITLG isoform, which promoted ERK phosphorylation and ultimately triggered the malignant proliferation of MM cells. Our findings on the mechanism of DAZAP1 promoting MM cell proliferation through AS provide new ideas and approaches for subsequent studies of aberrant AS in MM.

**Figure 7:**
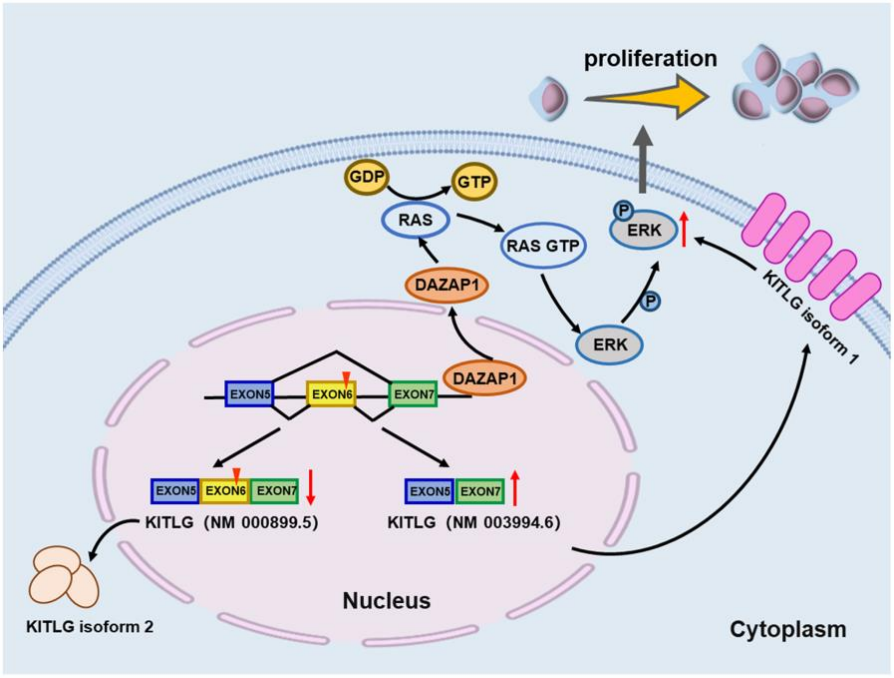
Schematic depiction illustrates that DAZAP1 promotes MM cell proliferation through alternative splicing of KITLG.

In summary, we demonstrate that DAZAP1 acts as a tumor-promoting gene with therapeutic potential. DAZAP1 plays a critical role in regulating the activation of ERK signaling pathway via modulating AS of *KITLG* mRNA to increase the proliferation of MM cells, which provide mechanistic insights into DAZAP1 as a promising therapeutic target for MM (Figure 7).

## MATERIALS AND METHODS

### Gene expression profiling

The gene expression profiles (GEP) were obtained from the GEO database. The MM GEP datasets used in this paper included GSE5900, total therapy 2 (TT2, GSE2658), total therapy 3 (TT3, GSE2658), the assessment of proteasome inhibition for extending remission (APEX, GSE9782), Trial Group for Hematology Oncology Group-65 (HOVON65, GSE19784), GSE136337, GSE38627 and GSE19554.

### Antibodies and reagents

Antibodies were used as follows: DAZAP1 (182558, Abcam, UK); FLAG (14793, Cell Signaling Technology, USA); GAPDH (60004-I-Ig, Proteintech, China); KITLG (sc-13126, Santa Cruz, USA); Puromycin was purchased from Merck KGaA (Darmstadt, Germany). Cell counting kit-8 (CCK8) was acquired from Apexbio, USA (K1018).

### Cell lines and cell culture

Human MM cell lines CAG and OCI-MY5 were cultured in RPMI-1640 (01-100-1ACS, Biological Industries, Israel). HEK293 cells were cultured in DMEM (01-052-1ACS, Biological Industries, Israel). All culture media were complemented with 10% fetal bovine serum (FBS, 04-001-1ACS, Biological Industries, Israel), 1% penicillin (100 U/mL, 03-031-1B, Biological Industries, Israel) and streptomycin (100 μg/mL, 03-031-1B, Biological Industries, Israel). All cells were cultured *in vitro* at 37°C in a humidified environment with 5% CO_2_.

### Plasmids and cell transfection

The plasmids containing the Homo DAZAP1 cDNA were designed and synthesized by Jinbeijin Biotechnology Co., Ltd. (Nanjing, China). The coding sequence of DAZAP1 was cloned into the lentiviral vector CD513B-1, which carried green fluorescence and flag tags. Plasmids were transformed into expression vector STBL3 competent cells and then picked an individual colony to amplify in LB media containing 50 μg/mL puromycin. It took about 8–12 h for plasmids to express in quantity in E. coli. Plasmid DNA was extracted from E. coli cultures using the EndoFree Mini Plasmid Kit (DP118, Tian Gen Biotech, Beijing, China). Lentiviruses containing the cDNA were obtained by co-transfection of the CD513B-1-DAZAP1 and packaging vectors (PLP1, PLP2 and PLP-VSVG) into HEK293 cells according to the manufacturer’s instructions of Lipofectamine Transfection Reagent. The virus supernatant was gathered after 48 h and was stored at −80°C for subsequent experiments. Wild-type MM cells were transfected with the lentiviruses retrieved above. Stably transfected cells were then selected by puromycin resistance.

### Transient transfection

Small interfering RNA (siRNA) was purchased from Feng Hui Biotechnology Co. (Changsha, China). The base sequences used to silence DAZAP1 were shown as follows: Sense strand (5′-3′): GCGAUAACAGUAAAUCAAAdTdT; Antisense strand (5′-3′): UUUGAUUUACUGUUAUCGCdTdT. The cells were resuspended with BTXpress electroporation solution to 10^6^/mL. Subsequently, siRNA was added into the solution to a final concentration of 100 nmol/L and then the solution was transferred into the BTX electroporation cuvettes plus. Two pulses for 1.0 s at 960 microF of capacitance, 200 V of voltage were the most favorable electrical parameters for EP efficiency.

### Western blotting (WB)

The prepared protein samples were separated by SDS-PAGE and transferred to PVDF membranes. The membranes were blocked with 5% skim milk before incubating primary antibody overnight at 4°C. The membranes were washed thoroughly and incubated for another 2 h with HRP-coupled secondary antibody (Santa, 1:1000). Proteins were detected applying ECL reagent (GE Healthcare Life Sciences) and images were captured by chemiluminescence imaging system.

### Cell proliferation and viability assay

Cell proliferation was assessed using the CCK-8 assay. The cells were seeded evenly in 96-well plates at a density of 10^3^ cells per well, and 10 μL of CCK8 was added to each well after 24 h, 48 h, 72 h, respectively. The cells were incubated at 37°C for additional 2 h before measuring the absorbance at 450 nm wavelength using a microplate reader.

### Soft agar colony formation

Cells were resuspended with RPMI 1640 medium containing 10% FBS and 1% penicillin/streptomycin solution to a density of 10^4^ cells/mL, and the above liquid was mixed with 0.33% agarose solution in a 1:1 ratio and spread in a 6-well plate. When the cell/agar mixture solidified, we placed the plate in a humidified incubator at 37°C. The medium was added twice a week to avoid drying of the cell/agar coagulate until colonies formed. Colonies were counted under a light microscope. In each experiment, cells were seeded in triplicate and three fields per well were quantified.

### GTP-RAS pull-down assay

RAS activity was detected using an Active RAS Pull-Down and Detection Kit (16117, Thermo, USA). Briefly, cell lysate was incubated with RBD-GST fusion proteins so that active RAS could be pulled down using glutathione-sepharose beads. Precipitates were washed 3 times and boiled in 50 μL of loading buffer for 5 min at 95–100°C to release bound proteins. Eluted proteins were separated on a 15% polyacrylamide gel, transferred to a PVDF membrane, and subjected to immunoblot analysis using anti-RAS antibodies.

### RNA immunoprecipitation sequencing

CAG EV and CAG DAZAP1-OE cells were lysed to release RBPs with bound RNAs, followed by incubating with the antibody-coated A/G Mag Beads. The beads were pulled off with a magnet and washed rigorously for several rounds to remove non-specific interactors. The beads were treated with proteinase K to isolate immunoprecipitated protein-bound RNA. Released RNA could be extracted by phenol-chloroform-isopropanol complexes. The obtained high quality RNAs were sequenced by the Illumina sequencing platform of Novo Gene Biotechnology Co., Ltd. (Beijing, China).

### AS analysis

The rMATS software was used to compare the results for AS classification and differential AS analysis, which could classify AS events into the following five categories and analyze differential AS in the samples with biological replicates [32, 33]. Five types of variable shearing events were defined as follows: SE (skipped exon), MXE (mutually exclusive exon), A5SS (alternative 5′ splice site), A3SS (alternative 3′ splice site), and RI (retained intron). The AS efficiency was quantified by inclusion level (PSI, percentage spliced in) and statistically analyzed by likelihood-ratio test. The splicing events with FDR (the corrected *p* value, the smaller the more credible) < 0.05 and | IncLevel1-IncLevel2 | > 0.05 were considered statistically significant. IncLevel was calculated based on the effective length of the isoform produced by the splicing event and the number of reads supporting the splicing event, and the result reflected the information on the average frequency of the respective exons contained in the final mRNA transcript of the sample. IncLevel1 represented the DAZAP1-OE group, and IncLevel represented the EV group.

### Quantitative PCR

Total RNA was extracted from MM cells using TRIeasy. One microgram of total RNA was reverse transcribed according to the manufacturer’s instructions by the Hifair 1st Strand cDNA synthesis super mix. Quantitative PCR (qPCR) was performed using SYBR Green Master Mix (Cat11201#, YEASEN, Shanghai). The primer sequence information was listed in Table 1. The qPCR system was initially predenatured at 95°C for 5 min, followed by 40 cycles of denaturation at 95°C for 10 sec, annealing at 60°C for 20 sec, elongation at 72°C for 20 sec. The threshold cycle values were standardized by the level of GAPDH mRNA. The specificity of the products was verified by melting curve analysis. Relative mRNA expression of the target genes was obtained by normalization to the control group and to GAPDH levels with the 2^−ΔΔCt^ method.

**Table 1:** Primers for qPCR.

**Target gene**	**Sequence**
KITLG (NM_000899.5) Forward primer	CAGAGTCAGTGTCACAAAACCATT
KITLG (NM_000899.5) Reverse primer	TTGGCCTTCCTATTACTGCTACTG
KITLG Forward primer	AGGAATCGTGTGACTAATAATG
KITLG Reverse primer	ACTTGGCTGTCTCTTCTT
GAPDH Forward primer	GGGGAGCCAAAAGGGTCATCATCT
GAPDH Reverse primer	GACGCCTGCTTCACCACCTTCTTG

### MM xenografted model

The 6~8-week old NOD/SCID mice were selected for subcutaneous injection of CAG EV and CAG DAZAP1-OE cells on the left and right side of the abdomen, respectively. The mice were euthanized until the tumor diameter reached 15 mm. Tumor tissues were dissected, photographed, and weighed. All animal experiments were carried out in compliance with the Recommendations for the Care and Use of Laboratory Animals and under the guidance of the Ethics Committee of Nanjing University of Chinese Medicine (Ethics Registration no. 201905A003).

### Statistical analysis

All data were expressed as mean ± SD. The statistical analyses were carried out using GraphPad Prism 8.0 version. Two-tailed Student’s *t*-test (2 groups) and one-way ANOVA (≥3 groups) were used to determine differences between experimental groups. Significance levels were set at *p* < 0.05. ^*^p < 0.05, ^**^p < 0.01 and ^***^p < 0.001.

## Supplementary Materials

Supplementary Figure 1
