# Supplementary material for: DAZAP1 facilitates the alternative splicing of KITLG to promote multiple myeloma cell proliferation via ERK signaling pathway
Source: Aging (Albany NY). 2022 Oct 13;14(19):7972–85. doi: 10.18632/aging.204326 (PMC9596219; doi:10.18632/aging.204326)
Supplement: Supplementary Figure 1 [file aging-14-204326-s001.pdf]

SUPPLEMENTARY FIGURE

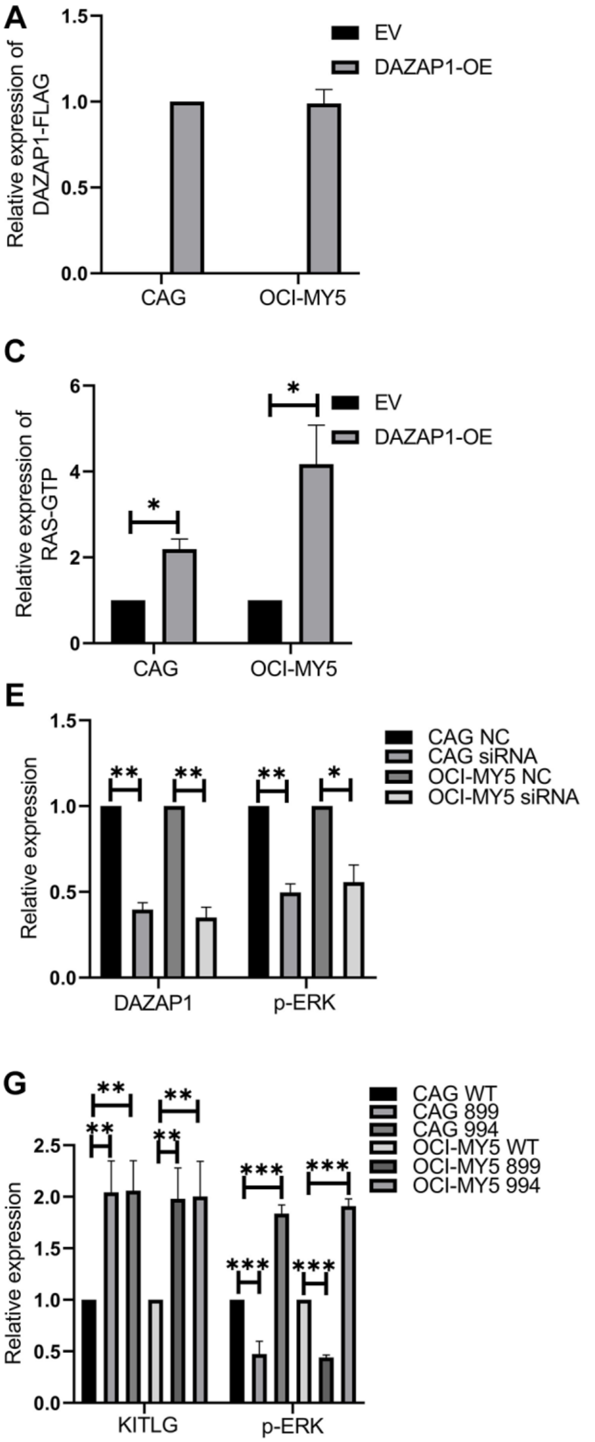

Supplementary Figure 1. The quantitative analyses of WB for the detection of the indicated protein expression. (**A** and **B**) Confirmation of DAZAP1 expression in DAZAP1-OE/siRNA MM cells. (**C**) Relative expression of RAS-GTP in EV and DAZAP1-OE MM cells. (**D** and **E**) Relative expressions of DAZAP1 and p-ERK in DAZAP1-OE and DAZAP1-KD MM cells, respectively. (**F** and **G**) Relative expressions of KITLG and p-ERK in HEK293, CAG and OCI-MY5 cells upon transfection with isoform 1 (994) and isoform 2 (899), respectively.
